# Risk Factors for Complications following Volar Locking Plate (VLP) Fixation of Unstable Distal Radius Fracture (DRF)

**DOI:** 10.1155/2022/9117533

**Published:** 2022-11-29

**Authors:** Yanbin Zhu, Song Liu, Yansen Li, Qianru Yang, Ruoxiang Miao, Yingze Zhang

**Affiliations:** ^1^Department of Orthopaedic Surgery, The Third Hospital of Hebei Medical University, Shijiazhuang, Hebei 050051, China; ^2^Key Laboratory of Biomechanics of Hebei Province, Shijiazhuang, Hebei 050051, China; ^3^Department of Foot and Ankle Surgery, The Third Hospital of Hebei Medical University, Shijiazhuang, Hebei 050051, China; ^4^Basic Medical College, Hebei Medical University, Shijiazhuang, Hebei 050000, China; ^5^Chinese Academy of Engineering, Beijing 100088, China

## Abstract

**Objective:**

To evaluate the incidence and risk factors for complications following volar locking plate (VLP) fixation of unstable distal radius fracture (DRF).

**Methods:**

This retrospective study identified patients who underwent VLP fixation of unstable DRF between 2017 and 2020 with a minimum 12-month follow-up assessments. By reviewing electronic medical records and follow-up notes, patients were categorized complication or noncomplication group. Differences in variables were detected by using univariate analyses, and independent factors were identified using multivariate logistic regression analysis.

**Results:**

During this study period, 423 patients were included, and 63 (rate, 14.9%) complications in 58 patients were documented. Wound infection (17, 4.0%) was most common, followed in decreasing frequency by carpal tunnel syndrome (13, 3.1%), tendon rupture/irritation (10, 2.4%), complex regional pain syndrome (8, 1.9%), and plate/screw-related complications (5, 1.7%). In the univariate analyses, 18 variables were found to be significantly different (*p* < 0.05). Logistic regression analysis identified 5 independent factors, including being male (OR, 3.5; *p* = 0.014), type C fracture (vs. type A, OR: 2.7, and *p* = 0.035), general anesthesia (vs. regional, OR: 2.4, and *p* = 0.045), bone grafting (OR, 6.3; *p* < 0.001), and surgery performed by less experienced surgeons (OR, 3.1; *p* = 0.003). The goodness-of-fit of the final model was acceptable.

**Conclusions:**

These factors will help surgeons individualize and stratify the risk of complications and help patients for risk counselling; especially, an informed clinical decision targeting those modifiable factors (anesthesia mode, bone grafting, and surgeon experience) can be considered, when indicated.

## 1. Introduction

Distal radius fracture (DRF) is among the most common fracture, accounting for about 1/6 of the skeletal fractures and presents with bimodal distribution, with peaks occurring at the adolescent and the elderly [1, 2]. Most DRFs can be treated via the nonoperative methods with reported favorable clinical outcome. However, for unstable DRFs, surgeries are consistently recommended in international expert consensus guidelines [3], and open reduction and internal fixation (ORIF) with volar locking plate (VLP) has become the mainstay of the treatment since its introduction [4, 5]. Despite that, the surgical outcomes were likely adversely affected by the high incidence of postoperative complications, which varied between 6% and 80% based on the study population and definitions of “complications” [6], and 20% to 40% necessitated secondary surgical procedures [7, 8]. Consequently, hospitalization stay was significantly prolonged, and total health-care costs were substantially increased, for example, a deep wound infection was associated with approximately 4-fold-increased cost of care (i.e., $23355 vs. $6383) [9].

The most cost-effective method is prevention, and for this purpose, identification of the risk factors, especially the modifiable ones to target them with effective interventions, is undoubtedly of paramount importance. In the past decade, a large body of researchers have identified a variety of risk factors for complications (overall or specific) following VLP fixation of DRF, e.g., comorbid diabetes [10], unhealthy lifestyles (e.g., smoking) [11–13], obesity [10, 14], injury characteristics (open fracture, severer fracture type, high-energy mechanism, and involvement of lunate facet) [13, 14], surgeon experience [14–16], surgical characteristics (placement position of plate relative to the volar rim according to Soong grading) [16, 17], longer operative time [13], residual intra-articular step-off [10]), and systemic condition (e.g., preoperative hypoalbuminemia [13]). Despite with more or less limitations (i.e., high selection of a specific population, ambiguous or confusing definitions of complications, or methodology of small sample, retrospective, and single-center design, and arbitrary definition), these findings do benefit surgeon and patients for preoperative counselling, individual risk assessment, and stratification.

However, to our best knowledge, many factors that were identified to influence adverse outcomes or complications following DRF surgery or other surgeries have not been well studied when specifying VLP procedures, for example bone grafting [18, 19]. In this study, we sought to identify some risk factors that potentially influenced the risk of complications following VLP fixation of DRFs.

## 2. Methods

### 2.1. Inclusion and Exclusion Criteria

This was a retrospective single-center study, and the study protocol was approved by the ethics committee of the 3^rd^ Hospital of Hebei Medical University (a tertiary referral hospital with a level I trauma center), which waived the requirement for participant-informed consent due to deidentified data used. Patients who underwent VLP fixation for acute unstable DRF between January 2017 and December 2020 were reviewed for inclusion. Inclusion criteria were age ≥ 18 years, unstable DRF stabilized by VLP within 21 days of injury, complete data on variables of interest, and a minimum of 12-month follow-up assessment. Exclusion criteria were open fracture, bilateral DRFs, other fractures in the ipsilateral upper limb, pathological or metastatic fractures, previous wrist surgery, treatments other than VLP, any concomitant dorsal plating or external fixation, missing data on variables, or inadequate follow-up assessments.

The operative indications were in accordance with the recommendations by the American Academy of Orthopaedic Surgeons (AAOS) guidelines: postreduction radial shortening > 3 mm, dorsal tilt > 10°, or intra-articular step-off or displacement > 2 mm [3].

### 2.2. Complications

Postoperative complications were available by inquiring the electronic medical records (EMR), picture archiving and communication system (PACS), outpatient visit notes, and the readmission EMR (via the unique identity number) for treatment of complications. The potential complications included wound infection (superficial or deep), carpal tunnel syndrome (CTS), symptomatic or painful hardware, complex regional pain syndrome (CRPS), malreduction and loss of reduction, plate/screw-related issues (plate loosening, screw penetration into articular surface, and too long screws causing symptoms), transient paresthesia, artery/nerve injury requiring injury, tendon irritation (extensor or flexor), tendon rupture (extensor or flexor), refracture, malunion, nonunion, and posttraumatic arthritis.

Tendon irritation and nerve irritation were defined based on patients' complaint of sore feeling when tendon was moving, hand or finger numbness or tingling, or the positive result of tapping or pressing test of nerve, respectively [20]. Delayed union was defined as incomplete healing (a lack of bony bridging) at 6 months and nonunion at 9 months after surgery, respectively [21]. Malunion was defined in accordance with the criteria proposed by AAOS guidelines based on final radiographs [3].

### 2.3. Variables of Interest

Two researchers (QY and RM) were responsible for collecting data on variables of interest by inquiring the EMR, PACS, operative notes, and laboratory reports. These variables included demographics (age, sex, educational attainment, place of residence, and occupation), cigarette smoking, alcohol drinking, comorbid conditions (hypertension, diabetes, obesity (body mass index ≥ 30 kg/m^2^), heart disease (chronic heart failure or coronary heart disease), chronic obstructive pulmonary disease (COPD), history of a cerebrovascular accident, liver disease (cirrhosis or hepatitis), and renal insufficiency), injury (fracture type based on AO classification, injury mechanism, affected side, and handedness), surgery (American Society of Anesthesiologists (ASA) classification, anesthesia mode, time to surgery, blood loss, operative time, use of bone graft, day of surgery (weekday or weekend/holiday), surgeon experience (number of VLP procedures since practice or within 12 months preceding the index procedure), and laboratory results upon admission (serum albumin, red blood cell, hemoglobin, hematocrit, white blood cell, neutrophils, lymphocytes, and glucose).

Low-energy injury was defined if a DRF was caused by a simple fall from a standing height; otherwise, e.g., fall from a height, motor accidents, and mechanical injuries were deemed as high-energy injury. Surgeons were classified as experienced if they performed ≥ 20 VLP of DRF procedure since practice, and otherwise, as less experienced. Meanwhile, during the study period, high-volume was defined if ≥7 VLP procedures were performed within 12 months preceding the index surgery and <7/year as low-volume. Bone graft included allogenous and autogenous bone graft, and in this study, we did not distinguish between them for statistical purpose because only a small proportion of patients were given bone graft. Weekend/holiday referred to Saturday, Sunday, and any officially issued holidays in China.

### 2.4. Statistical Analysis

Two-step strategy was used for statistical analysis. First, we performed the bivariate analysis to compare patient demographics, injury, comorbidities, lifestyles, surgery, and laboratory results at admission, using the Student *t*-test or Mann–Whitney *U*-test for continuous variables and Pearson chi-squared test or Fisher' exact test for categorical variables, when appropriate.

Second, variables tested with *p* < 0.10 in the univariate analyses were entered into the multivariate logistic regression analysis by using stepwise backward selection. In the final model, variates with *p* < 0.10 were retained. The magnitude of association between variables and outcome (overall complications) was indicated by odd ratio (OR) with 95% confidence interval (95% CI). Hosmer-Lemeshow test was used to evaluate the goodness-of-fit of the final modal, with *p* > 0.05 representing an acceptable result; also, adjusted Nagelkerke R^2^ was used to quantify the model fit degree, with larger value indicating better fit. Statistical significance was set as 2-tailed *p* < 0.05. SPSS26.0 (IBM Corporation, Armonk, New York, USA) was used to perform all the analyses.

## 3. Results

### 3.1. General Characteristics

During the study period, 423 patients met the inclusion criteria and were included, including 244 males and 179 females, with an average age of 46.0 ± 13.5 (range, 18-79 years). Over 80% occurred in patients younger than 60 years. Fractures occurred in the left wrist in 197 (46.6%) and right in 226 (53.4%) patients and dominant wrist in 240 (56.7%) and nondominant wrist in 183 (43.3%) patients. Based on AO classification, there were 140 (33.1%) type A, 93 (22.1%) type B, and 190 (44.9%) type C fractures. The VLP procedure was performed at a mean of 6.5 ± 5.0 days after injury, with a range of 0-21 days. There were 39 surgeons (21 hand surgeons and 18 orthopaedic trauma surgeons) performing the procedures, with 13.2 per surgeon. The 12 experienced surgeons (≥20 since practice) accounted for the 298 procedures and the 27 less experienced surgeons for the remaining 125 cases. About 62% of the procedures were performed by high-volume surgeons (≥7 proceeding the index surgery).

### 3.2. Overall and Specific Complications

There were 63 cases of complications in 58 patients, representing an overall rate of 14.9% (63/423). The most common complication was wound infection (17, 4.0%), including 13 (3.1%) superficial wound infection which resolved by dressing changes and oral antibiotics, and 4 (0.9%) deep wound infection which was treated by intravenous antibiotics and/or surgical debridement in 3 and early removal of hardware in 1 case. Of note, 82.4% (14/17) were early onset infection (<2 weeks), and 3 (17.6%) were delayed (2-10 weeks), without late infection (>10 weeks), based on the previously proposed classification for surgical site infection [22].

The second commonest complication was CTS, representing an incidence rate of 3.1% (13/423); among them, 2 were acute and resolved by concomitant carpal tunnel release along with VLP fixation. The remaining 11 occurred after VLP, resolving by conservative management in 3 cases and by carpal tunnel release in 8 cases. Other nerve-related complications included palmar sensory branch of the median nerve in 2 (0.5%) cases after trauma and 2 (0.5%) caused by surgical trauma.

Tendon rupture/irritation occurred in 10 (2.4%) patients, with most involving extensor tendon (1.7%, 7/423; extensor pollicis longus, 4; extensor carpi radialis brevis/longus, 2; extensor digitorum communis, 1) and flexor tendon in 3 (0.7%, 3/423). CRPS was reported in 8 (1.9%) patients and wrist stiffness in 3 (0.7%) patients, which were relieved by strengthened physical and rehabilitation therapy, and in some severe cases, with combined pharmacologic treatment. Plate/screw-related complications (5 cases, 1.7%) encountered in this study were screw penetrated into the joint surface by in 3 and two long screw causing symptomatic pain in 2 patients, without plate/screw breakage encountered. The other complications included delayed union in 2, nonunion in 2, iatrogenic radial artery requiring repair in 1, and radiocarpal arthritis demonstrated as radiographs.

### 3.3. Univariate and Multivariate Analyses

The univariate analysis showed the significant difference between patients with and without complications in terms of sex distribution (male, 77.6% vs. 57.5%, *p* = 0.004), living place (rural, 75.9% vs. 60.8%, *p* = 0.028), injury mechanism (high-energy, 37.9% vs. 23.3%, *p* = 0.017), fracture type based on AO classification (*p* = 0.016), ASA classification (*p* = 0.009), anesthesia mode (general, 34.5% vs. 18.4%, *p* = 0.005), surgical duration in continuous (170.3 ± 94.9 vs. 132.1 ± 66.9, *p* = 0.004) and in categorical variable (≥180 mins, 39.7% vs. 20.5%, *p* < 0.001), surgeon experience (experienced, 36.2% vs. 75.6%, *p* < 0.001), date for surgeries (24.1% vs. 12.9%, *p* = 0.023), bone grafting (20.7% vs. 6.0%, *p* < 0.001), allogeneic blood transfusion (25.9% vs. 7.9%, *p* < 0.001), and albumin either in continuous (30.3 ± 6.7 vs. 40.8 ± 5.2 g/L, *p* = 0.002) or categorical variable (<35 g/L, 29.3% vs. 14.2%, *p* = 0.004), as well as WBC count (10.0 ± 4.3 vs. 8.8 ± 2.6, *p* = 0.003; >10^∗^10^9^/L, 41.4% vs. 28.2%, *p* = 0.042), RBC count (4.0 ± 0.7 vs. 4.2 ± 0.6^∗^10^12^/L, *p* = 0.013; < lower limit, 29.3% vs. 12.6%, *p* = 0.001), hemoglobin (121.8 ± 20.2 vs. 130.5 ± 19.2, *p* = 0.002; < lower limit, 31.0% vs. 14.5%, *p* = 0.002) and hematocrit (36.3 ± 5.9 vs. 38.7 ± 5.7, *p* = 0.003; < lower limit, 44.8% vs. 23.8%, *p* = 0.001), and neutrophil count (7.4 ± 4.0 vs. 6.4 ± 2.5, *p* = 0.009) and sodium concentration in continuous variable (138.1 ± 3.4 vs. 139.0 ± 3.3, *p* = 0.033) (Table 1).

The multivariate logistic regression analyses showed that male sex (OR, 3.5; 95% CI, 1.3 to 9.8), type C vs. type A fracture (OR, 2.7; 95% CI, 1.2 to 5.8), general vs. regional anesthesia (OR, 2.4; 95% CI, 1.0 to 5.6), bone grafting (OR, 6.3; 95% CI, 2.5 to 16.3), and surgeries performed by less experienced surgeons (OR, 3.1; 95% CI, 1.6 to 5.2) were significantly associated with increased risk of complications, and the detailed results were listed in Table 2. The Hosmer-Lemeshow test showed the acceptable goodness-of-fit of the final model (*X*^2^ = 7.294, *p* = 0.505, and Nagelkerke *R*^2^ = 0.385).

## 4. Discussion

The knowledge of the postoperative complications would help optimize the care of the patients and provide necessary information on prognosis for both surgeons and patients. Despite the plentiful literature on DRF treatment and outcome, studies specifying at complications following VLP fixation are still lacking. This study identified the overall incidence rate of complications of 14.9% and 5 factors associated with the increased risk of complications, including male sex, type C fracture (vs. type A), general anesthesia, bone grafting, and surgeries performed by less experienced surgeons.

The incidence of complications varied greatly following VLP fixation of DRF in literature, ranging from 4% to 36%, primarily attributable to study population, methodology, and follow-up periods [23–25]. Besides, the variables definition of “complication” played a particular role. In a systematic review, McKay et al. [6] found a greatly varied overall rate of 6% to 80%; even for a specific complication, e.g., posttraumatic arthritis, it varied from 7% to 65%. In their view, concomitant or associated injury should be defined as “diagnoses” rather than “complications,” because they were not a result of DRF or treatment. Meanwhile, not all suboptimal results should be treated as “complications,” e.g., wrist stiffness should be considered as a “symptom,” unless it was attributable to a specially diagnosed complication, such as radiocarpal arthritis [6]. Therefore, the median rate observed in this study should be interpreted in our specific settings, and standardization of definition of complications to develop a checklist is warranted to facilitate homogeneous comparisons and thus improve the care of DRF.

Previously, being female was reported to be associated with higher levels of residual pain, disability, and function [26–28], which was largely attributable to the predominance of females with osteoporosis for DRFs. In this study, being male was found to be 3.5 times increased risk of complications. Rather than the biologic and hormonal effects, this result might be more likely explained by the high-impaction trauma leading to the severer fracture and associated tissue injuries and the resultant more surgical trauma for management.

Consistent with previous findings, less experience would adversely affect the surgical prognosis, primarily due to that VLP was technically demanding and had a steep learning curve. Ward et al. retrospectively reviewed 92 patients with 96 DRFs treated by VLP and found the first 30 patients experienced significantly more complications than did the later 62 patients (37% vs. 17%, *p* = 0.03), concluding that a considerable proportion of complications was avoidable. In Sirnio et al.'s study, authors identified low-volume surgeon (<20 vs. ≥20 VLP procedures) as an independent risk factor for plate-related complications. However, this observation was not always consistent. Soong et al. [29] found more late complications with surgeons with experience of >20 procedures, attributing it to the variable diagnosis of tendon irritation which accounted for the most common late complications.

In this study, type C fracture was associated with 2.7-fold-increased risk of complications, partly due to higher degree of trauma from fracture and surrounding soft tissues. Especially for type C2 and C3 fractures, articular surface comminution, lunate facet impaction, and radial shortening were generally accompanied, which required advanced surgical skill to reduce and stabilize to maintain until to bone union [30]. We suggest that treatment of type C2/C3 fractures should be centralized to specialized hand surgeons, if not, to orthopaedic or trauma surgeons under supervision of a consultant surgeon.

The effects of regional versus general anesthesia on complications after DRF surgery have been defined by high-level-evidence studies. By using a randomized trial design, Galos et al. [31] found the only benefits of postoperative 12-24 hr pain control for general anesthesia compared to regional anesthesia but not for any functional outcome scores at 6 or 12 weeks. More recently, Lee et al. [18] demonstrated almost the same safety profile in terms of any complication, minor complication, major complication, unplanned reoperation, unplanned readmission, and mortality (all ≥0.083) between regional and general anesthesia technique within 30 days after ORIF of DRFs, using the ACS-NSQIP cohort of over 10,000 DRF patients and an advanced matching methodology based on coarsened-exact-matching (CEM). The authors concluded that regional anesthesia might be the preferred anesthetic technique in “patients at high risk with general anesthesia, such as those with reactions to general anesthesia in the past or with significant cardiopulmonary risk factors” [18]. In this study, we found the significantly more complications associated with general anesthesia, primarily due to our applied management strategy that patients with complex medical conditions or injury would be cared with general anesthesia for safety purpose, more likely suggesting general anesthesia as a proxy for complex medical conditions or injury.

We found a surprisingly increased risk of complications by 6.3-times associated with bone grafting, and of note, we did not distinguish between autogenous and allogenous bone grafting for statistical purpose. There were several explanations for this “seeming surprising” result. First, this more likely reflected the greater severity of fracture itself, e.g., fracture comminution and multiple small-size fragments unable to reduce, thus leaving bone void for filling after reduction. Second, this reflected the more surgical trauma from, e.g., skin incision, soft tissue dissection, and prolonged-time for tissue traction for reduction and autogenous bone graft harvesting and filling, resulting increased risk of tissue ischemia and hypoxia. Third, the risk of bioincompatibility and immunological rejection of allogenous bone graft or substitute, together with the relatively poor revascularization [32], should be considered as an important role in wound infection.

Despite multiaspect confounders for adjustment and a relatively large sample size, several limitations to this study should be noted. First, the inherent limitation was the retrospective design, which might have biased the accuracy of data collection. Furthermore, the self-reported comorbidities or conditions could not be objectively verified. Secondly, one of important reasons for greatly varied complication rates reported in literature was the nonstandardization of definitions, and this concern remained in this study. For some, minor complications, such as transient paralysis or superficial wound infection that easily resolved by oral antibiotics, whether they were documented in medical records or follow-up notes, were more likely at the treating surgeon's discretion, based on their experience and expertise. Third, our findings of SSI being the most frequent complication were more likely related to extent of damage to the surrounding soft tissue or the surgical trauma, but relevant data were unavailable or unable to be quantified. In addition, postoperative exercises (initiation, duration, intensity, and frequency), operation room arrangement, or staff experience may also have contributed to the odd of complications, which were either not available. Therefore, the residual confounding effects remain. Fourth, the relationship identified between factors and complications was associative rather than causative and should be treated cautiously when interpreting. Fourth, this was a single-center study in a tertiary referral institution with a level I trauma center more likely with severer fracture or complicated medical conditions, thus not representing the average level for treating this injury.

In summary, the overall incidence of complication following VLP fixation of DRF was 14.9%, and 5 factors (including 3 modifiable ones) were identified as independently associated with complications. These factors will help surgeons individualize and stratify the risk of complications and help patients for risk counselling; especially, a more informed clinical decision targeting those modifiable factors (anesthesia mode, bone grafting, surgeon experience, and surgical date) can be made, when indicated, ultimately improving the prognosis of DRF. The future prospective, multicenter studies are warranted to validate our findings.

## Figures and Tables

**Table 1:** Comparisons of between patients with and without complications by univariate analysis.

Variables	Number (%) of patients without complications (*n* = 365)	Number (%) of patients with complications (*n* = 58)	*p*
Age (years)	46.4 ± 13.5	43.4 ± 13.3	0.117
(i) 18-39	129 (35.3)	20 (34.5)	0.224
(ii) 40-59	160 (43.8)	31 (53.4)	
(iii) ≥ 60	76 (20.8)	7 (12.1)	
Sex (males)	210 (57.5)	45 (77.6)	0.004
Occupation (manual work)	262 (71.8)	47 (81.0)	0.140
Living place (rural)	222 (60.8)	44 (75.9)	0.028
Educational attainment (≥ 12 years)	92 (25.2)	11 (19.0)	0.304
BMI (kg/m^2^)	25.2 ± 3.5	24.7 ± 3.0	0.336
(i) ≥ 28	74 (20.3)	7 (12.1)	0.140
Hypertension	45 (12.3)	11 (19.0)	0.166
Diabetes mellitus	96 (26.3)	12 (20.7)	0.363
Heart disease	26 (7.1)	6 (10.3)	0.389
Cerebrovascular disease	16 (4.4)	4 (6.9)	0.402
COPD	18 (4.9)	4 (69)	0.531
Liver diseases	10 (2.7)	0 (0.0)	0.202
Renal insufficiency	4 (1.1)	0 (0.0)	0.423
Current smoking	77 (21.1)	12 (20.7)	0.944
Alcohol drinking	122 (33.4)	22 (37.9)	0.501
High-energy injury	85 (23.3)	22 (37.9)	0.017
Involvement of right hand	197 (54.0)	29 (50.0)	0.573
Involvement of dominant hand	209 (57.3)	31 (53.4)	0.586
Fracture type (based on AO classification system)			0.016
(i) A	128 (35.1)	12 (20.7)	
(ii) B	83 (22.7)	10 (10)	
(iii) C	154 (42.2)	36 (62.1)	
Time from injury to operation (days)	6.4 ± 4.6	7.6 ± 6.9	0.168
(i) < 7	253 (69.6)	33 (56.9)	0.055
(ii) ≥ 7	111 (30.4)	25 (43.1)	
ASA classification			0.009
(i) I	50 (13.7)	8 (13.8)	
(ii) II	278 (76.2)	36 (62.1)	
(iii) III-IV	37 (10.1)	14 (24.1)	
Anesthesia mode			0.005
(i) Regional	298 (81.6)	38 (65.5)	
(ii) General	67 (18.4)	20 (34.5)	
Surgical duration (minutes)	132.1 ± 66.9	170.3 ± 94.9	0.004
(i) ≥ 180	75 (20.5)	23 (39.7)	<0.001
Surgeries performed by experienced surgeons	276 (75.6)	21 (36.2)	<0.001
High-volume surgery (≥ 7 procedures within 12 months preceding the index surgery)	232 (63.6)	31 (53.4)	0.140
Surgeries performed at weekend/holiday	47 (12.9)	14 (24.1)	0.023
Bone grafting	22 (6.0)	12 (20.7)	<0.001
Allogeneic blood	29 (7.9)	15 (25.9)	<0.001
Albumin level (g/L)	40.8 ± 5.2	30.3 ± 6.7	0.002
(i) < 35	52 (14.2)	17 (29.3)	0.004
Sodium concentration (mmol/L)	139.0 ± 3.3	138.1 ± 3.4	0.033
(i) < 135	37 (10.1)	8 (13.8)	0.402
Glucose	5.8 ± 1.3	5.9 ± 1.4	0.419
(i) > 6.1 mmol/L	98 (26.8)	17 (29.3)	0.696
WBC (^∗^10^9/^L)	8.8 ± 2.6	10.0 ± 4.3	0.003
(i) > 10	103 (28.2)	24 (41.4)	0.042
Neutrophil count (^∗^10^9^/L)	6.4 ± 2.5	7.4 ± 4.0	0.009
(i) > 6.3	176 (48.2)	31 (53.4)	0.459
Lymphocyte count (^∗^10^9^/L)	1.6 ± 0.6	1.5 ± 0.5	0.189
(i) < 1.8	66 (18.1)	11 (19.0)	0.871
RBC (^∗^10^12^/L)	4.2 ± 0.6	4.0 ± 0.7	0.013
(i) < Lower limit	46 (12.6)	17 (29.3)	0.001
Hemoglobin (g/L)	130.5 ± 19.2	121.8 ± 20.2	0.002
(i) < Lower limit	53 (14.5)	18 (31.0)	0.002
Hematocrit (%)	38.7 ± 5.7	36.3 ± 5.9	0.003
(i) < Lower limit	87 (23.8)	26 (44.8)	0.001

Abbreviations: BMI: body mass index; COPD: chronic obstructive pulmonary disease; ASA: American Society of Anesthesiologists; WBC: white blood cell; RBC: red blood cell. Notes: RBC, reference range: female, 3.5 − 5.0^∗^10^12^/L; males, 4.0 − 5.5^∗^10^12^/L; reference range for hemoglobin: females, 110-150 g/L and males, 120-160 g/L; reference range for hematocrit: females, 35%-45%; males, 40%-50%.

**Table 2:** Multivariate analysis identifying 5 factors independently associated with complications after VLP fixation of DRF.

Variables	OR and 95% CI	*p*
Sex (male vs. female)	3.5 (1.3 to 9.8)	0.014
Fracture type		
(i) A	Reference	
(ii) B	1.2 (0.5 to 2.7)	0.344
(iii) C	2.7 (1.2 to 5.8)	0.035
Anesthesia mode (general vs. regional)	2.4 (1.0 to 5.6)	0.045
Bone grafting	6.3 (2.5 to 16.3)	<0.001
Surgery performed by less experienced surgeon (vs. experienced surgeon)	3.1 (1.6 to 5.2)	0.003

## Data Availability

All the data will be available upon motivated request to the corresponding authors of the present paper.

## Abbreviations

VLP: Volar locking plate
EMR: Electronic medical records
PACS: Picture archiving and communication system
AO: Arbeitsgemeinschaftfür Osteosynthesefragen
COPD: Chronic obstructive pulmonary disease
CTS: Carpal tunnel syndrome
CRPS: Complex regional pain syndrome
BMI: Body mass index
ASA: American Society of Anesthesiologists
SD: Standard deviation
OR: Odd ratio
WBC: White blood cell
RBC: Red blood cell.
